# Supplementary figures and images for: Development of Triangle RNA Nanostructure for Enhancing RNAi-Mediated Control of Botrytis cinerea Through Spray-Induced Gene Silencing Without Extra Nanocarrier
Source: Biology (Basel). 2025 Nov 18;14(11):1616. doi: 10.3390/biology14111616 (PMC12650204; doi:10.3390/biology14111616)

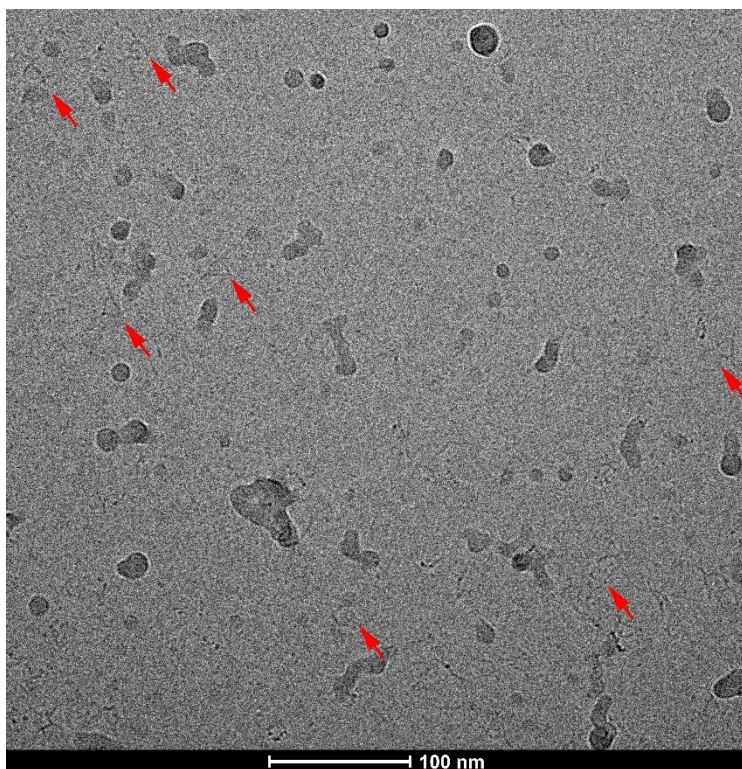

**Figure S1.** Bc-triangle cryo-EM Results.

Supplement: Supplementary file 1 [file biology-14-01616-s001.zip › biology-3915191-supplementary.pdf]
